# Facts and Hopes on RAS Inhibitors and Cancer Immunotherapy

**DOI:** 10.1158/1078-0432.CCR-22-3655

**Published:** 2023-08-15

**Authors:** Jesse Boumelha, Miriam Molina-Arcas, Julian Downward

**Affiliations:** 1 Francis Crick Institute, London, United Kingdom.

## Abstract

Although the past decade has seen great strides in the development of immunotherapies that reactivate the immune system against tumors, there have also been major advances in the discovery of drugs blocking oncogenic drivers of cancer growth. However, there has been very little progress in combining immunotherapies with drugs that target oncogenic driver pathways. Some of the most important oncogenes in human cancer encode RAS family proteins, although these have proven challenging to target. Recently drugs have been approved that inhibit a specific mutant form of KRAS: G12C. These have improved the treatment of patients with lung cancer harboring this mutation, but development of acquired drug resistance after initial responses has limited the impact on overall survival. Because of the immunosuppressive nature of the signaling network controlled by oncogenic KRAS, targeted KRAS G12C inhibition can indirectly affect antitumor immunity, and does so without compromising the critical role of normal RAS proteins in immune cells. This serves as a rationale for combination with immune checkpoint blockade, which can provide additional combinatorial therapeutic benefit in some preclinical cancer models. However, in clinical trials, combination of KRAS G12C inhibitors with PD-(L)1 blockade has yet to show improved outcome, in part due to treatment toxicities. A greater understanding of how oncogenic KRAS drives immune evasion and how mutant-specific KRAS inhibition impacts the tumor microenvironment can lead to novel approaches to combining RAS inhibition with immunotherapies.

## Introduction

The RAS family of genes are among the most frequently mutated genes in human tumors, being altered in up to 20% of all cancers (1). Of the three main isoforms that are found to be mutated in cancer—KRAS, HRAS, and NRAS—KRAS is the most commonly mutated isoform, present in up to 90% of pancreatic cancer (PDAC), 50% of colorectal cancer, and 30% of lung cancer (LUAD). RAS proteins are small guanine nucleotide binding proteins (GTPases) that act as signaling hubs coordinating the activity of multiple downstream signaling pathways (2) including those involved in cell proliferation, migration, survival, and metabolism. In response to mitogens, RAS proteins cycle between an inactive GDP-bound state and an active GTP-bound state, which can bind downstream effector proteins. Oncogenic RAS mutations impair GTP hydrolysis, which stabilizes the protein in the active GTP-bound form, resulting in constitutive mitogen-independent downstream effector signaling. These mutations principally arise as single amino acid substitutions in codons G12, G13, or Q61 (1).

Given the high frequency of RAS mutations in human cancer, there has been an extensive effort over the past three decades to pharmacologically target the RAS signaling pathway, which until recently has proved largely unsuccessful. Unlike protein kinases which have come to be seen as mostly successfully druggable, RAS proteins lack deep hydrophobic pockets and also bind GTP at picomolar affinity. Initial approaches to target RAS therefore focused on inhibiting the posttranslational modifications required to localize RAS to the plasma membrane, which is required for its biological activity. Farnesyltransferase inhibitors (FTI) were developed that prevent addition of a farnesyl lipid to the C-terminus of RAS, which is required for association with the plasma membrane. However, these inhibitors failed to show any benefits in clinical trials involving KRAS-mutant cancers (3). Although efficient at preventing the localization of HRAS to the plasma membrane, it was later shown that KRAS and NRAS can be alternatively prenylated by geranylgeranyltransferases. However, FTIs have recently been shown to have promising clinical activity in HRAS-driven head and neck squamous carcinoma (4).

Subsequent efforts have involved targeting RAS effector proteins, which have been shown using elegant genetic mouse models to be required for the maintenance of RAS-mutant cancer (5, 6). A plethora of inhibitors have been successfully developed targeting numerous RAS effector proteins, primarily kinases within the RAF/MEK/ERK and PI3K/AKT/mTOR pathways. Such drugs often have potent therapeutic activity in preclinical models; however, they have since been shown to have limited activity in clinical trials (7, 8). This has been attributed to the extensive signaling redundancy that exists in the RAS signaling pathway, multiple feedback mechanisms which causes pathway reactivation, and also narrow therapeutic window as the pathways are required in normal tissue. Combinations of therapies designed to address pathway redundancy and feedback mechanisms have been seriously hampered by high toxicities observed in clinical trials (9, 10).

## Development of Clinical RAS Inhibitors

Given the failures associated with targeting downstream effectors of RAS, developing direct inhibitors of RAS remains an attractive approach. Efforts led by Kevan Shokat resulted in a remarkable breakthrough in 2013 with the development of covalent allele-specific inhibitors targeting KRAS G12C (11). This approach exploited the reactivity of the cysteine substitution, which can be irreversibly targeted by a thiol-reactive warhead. These inhibitors specifically bind to GDP-bound KRAS G12C, trapping the protein in an inactive state and preventing downstream effector interaction. This is effective as, unlike other KRAS mutants, KRAS G12C has similar intrinsic GTPase activity compared with wild-type KRAS and therefore spends a significant proportion of its life cycle in a GDP-bound state. Importantly, the mutant-specific nature of these inhibitors enables inhibition of KRAS signaling in KRAS G12C mutant cancer cells while sparing signaling in normal cells, thereby widening the therapeutic window compared with nonmutant selective inhibitors (12, 13).

KRAS G12C mutations occur most often in lung cancer, accounting for nearly 50% of KRAS-mutant LUAD. Accordingly, clinical assessment of KRAS G12C inhibitors has focused initially on patients with metastatic lung cancer. Two clinical compounds, sotorasib (AMG 510) and adagrasib (MRTX849) have recently been approved by the Federal Drug Administration for the treatment of patients with previously-treated advanced non–small cell lung cancer (NSCLC). Both drugs achieved a favorable objective response rate (ORR) of 37% to 45% with moderate toxicities (20%–45% grade 3 or higher) in phase II clinical trials (14, 15). Similarly, both sotorasib and adagrasib have shown promising clinical activity in KRAS G12C PDAC (16), although these mutations are rare in this tumor type. Single-agent activity is much reduced in colorectal cancer, but this has recently been improved by combining with the EGFR inhibitor cetuximab (17).

Although KRAS G12C inhibitors have demonstrated impressive clinical activity, as seen with other targeted therapies, responses are often short-lived as resistance invariably arises in the majority of patients. Indeed, results from the recent phase III clinical trial comparing the efficacy of sotorasib with docetaxel, while demonstrating increased progression-free survival, failed to improve the overall survival of patients with lung cancer (18). Numerous preclinical and clinical studies have begun shedding light upon mechanisms underlying resistance to KRAS G12C inhibitors, which include mutations in KRAS that inhibit drug binding (19), activating mutations in downstream signaling components (20) and resynthesis of new KRAS G12C, which is maintained in a GTP-bound state by upstream receptor tyrosine kinase signaling (21). A number of clinical trials combining KRAS G12C inhibitors with other therapies, including chemotherapy and targeted therapy, are currently underway. However, genetic profiling of resistant tumors has demonstrated that patients often contain multiple resistant-conferring mutations (19) and therefore the feasibility of any single combination strategy to overcome acquired resistance remains unclear.

Because the identification of the druggable allosteric pocket of KRAS G12C 10 years ago, impressive progress has been made in the identification of other KRAS inhibitors besides the initial drugs targeting the inactive form of KRAS G12C. These include inhibitors targeting the active form of KRAS G12C or against other KRAS mutant proteins such as G12D, G12S, and G12R (22–24). The KRAS G12D inhibitor from Mirati Therapeutics MRTX1133 recently entered clinical trials. This noncovalent compound targets both the inactive and active form of KRAS G12D, the most common KRAS mutation in PDAC and colorectal cancer. Revolution Medicines have taken a different approach, developing covalent tri-complex inhibitors that target the active form of RAS, with their multi-RAS and KRAS G12C inhibitor already in clinical trials. More recently, Boehringer Ingelheim has reported the development of a noncovalent KRAS inhibitors, which binds preferentially to the inactive form and targets both wild-type KRAS and a broad range of KRAS mutant forms (25).

## Oncogenic KRAS Signaling Suppresses Antitumor Immunity

**Figure 1.**
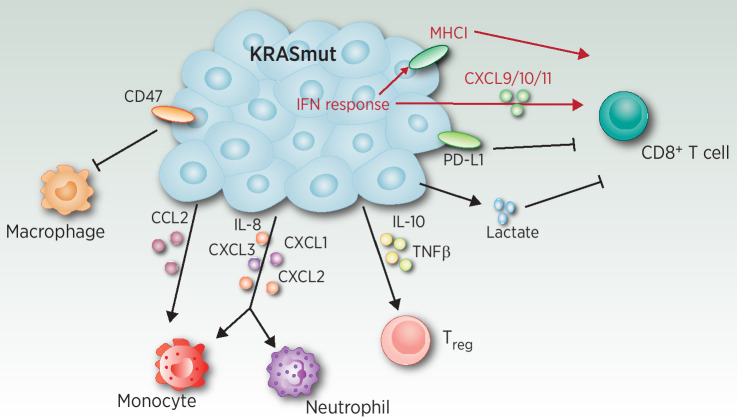
Mechanisms by which oncogenic KRAS drives immune evasion. Oncogenic KRAS can modulate the response of immune cells by different mechanisms, including secretion of cytokines, chemokines, and metabolites or expression of membrane proteins. Black text and lines indicate mechanisms activated by KRAS, and red indicates inhibition. Arrows represent enhancement of the immune cells and flat heads represent inhibition.

Decades of research have focused on how oncogenic mutations drive cell-autonomous processes that contribute to tumorigenesis, which has led to the development of a plethora of targeted therapy agents. However, it has become increasingly apparent that oncogenic signaling can extend beyond the cancer cell-intrinsic compartment and engage with the host immune system (26). Oncogenic KRAS has long been known to drive the expression of numerous cytokines and chemokines that promote an immunosuppressive tumor microenvironment (TME; Fig. 1). This was first demonstrated in a study, which identified IL8 as a direct transcriptional target of KRAS that promoted tumor-associated inflammation and tumor progression (27). Numerous other CXCR2 ligands have since been shown to be highly expressed in preclinical models of KRAS-mutant cancer, including CXCL1, CXCL2, CXCL3, and CXCL5 (28–30). The expression of CXCR2 ligands leads to the recruitment of immunosuppressive neutrophils and monocytes, which can suppress antitumor immunity. Indeed, either genetic or pharmacologic inhibition of CXCR2 has been shown to reverse immunosuppression and hinder tumor progression in KRAS-mutant mouse models (28, 30). Beyond CXCR2 ligands, oncogenic KRAS promotes the secretion of other myeloid chemoattractants such as CCL2, which promotes the migration of monocytes from the bone marrow into the TME (31). Oncogenic KRAS has also been shown to induce GM-CSF in pancreatic cancer, resulting in the expansion of immunosuppressive Gr1^+^CD11b^+^ myeloid cells and inhibition of T-cell–mediated tumor control (32). Furthermore, KRAS can directly inhibit the phagocytic capacity of antitumorigenic macrophages by driving tumor cell expression of the “do not eat me” myeloid checkpoint molecule CD47 (33). PI3K pathway signaling represses the expression of the CD47-targeting miRNA, miR-34a, resulting in increased CD47 expression and impaired macrophage phagocytosis. Indeed, KRAS-mutant LUAD tumors have increased expression of CD47.

KRAS signaling also dampens the function of tumor-infiltrating T cells via numerous mechanisms. The MAPK pathway has been shown to directly induce the expression and secretion of IL10 and TGFβ, which promote the conversion of conventional Th1 CD4^+^ T cells into immunosuppressive regulatory T cells (Tregs; ref. 34). Numerous studies have demonstrated the importance of Tregs in preclinical models of KRAS-mutant lung cancer as depletion of these immunosuppressive cells stimulates antitumor immunity and reduces tumor growth (35). Oncogenic KRAS can also directly inhibit cytotoxic T-cell responses by promoting tumor cell expression of the immune checkpoint ligand PD-L1 (36). PD-L1 mRNA contains AU-rich elements in the 3′ UTR, which promote transcript degradation via the RNA-binding protein tristetraprolin (TTP). MAPK pathway signaling results in phosphorylation and inhibition of TTP resulting in stabilization of PD-L1 mRNA. These support clinical observations that demonstrate increased PD-L1 expression in KRAS-mutant NSCLC tumors (37). KRAS signaling can reprogram tumor cell metabolism to support tumor growth, however this can also influence the composition of metabolites in the TME, which has a major influence on immune cell function. In colorectal cancer, oncogenic KRAS drives the production of lactic acid, leading to its accumulation in the TME whereby it promotes activation-induced cell death of tumor-specific CD8^+^ T cells (38).

Numerous immunosuppressive pathways have been shown to be induced downstream of the MAPK or PI3K signaling pathways, however oncogenic KRAS also activates the pro-inflammatory NF-κB pathway which induces the expression of numerous cytokines and chemokines. Downstream of KRAS, RAL proteins directly activate the kinase TBK1 (39), which in complex with IKKε drives the activation of NF-κB. This pathway can drive the secretion of IL6 in KRAS-mutant lung cancer (40). Indeed, pharmacologic blockade of IL6 in a mouse model of KRAS-mutant lung cancer results in a reduction in protumorigenic macrophages and Tregs with concomitant activation of CD8^+^ T cells (41).

Oncogenic KRAS has also been shown to repress tumor-intrinsic IFN signaling in lung cancer (31), colorectal cancer (28), and pancreatic cancer (42). Tumor-intrinsic IFN signaling plays an important role in immunosurveillance as it promotes MHC-I antigen presentation and the secretion of T-cell chemoattractants. The molecular mechanism underlying KRAS-mediated repression of IFN signaling has been shown to be mediated by MYC (31), which can directly inhibit the expression of numerous IFN pathway genes (42). This mechanism is consistent with the well-established idea that KRAS and MYC co-operate to drive tumorigenesis. Indeed, another study demonstrated that co-activation of MYC in KRAS-mutant lung tumors reshapes the TME via secretion of CCL9 and IL23, which results in the exclusion of T cells, B cells, and NK cells and infiltration of immunosuppressive M2 macrophages (43).

KRAS mutant lung cancer is frequently co-mutated with the tumor suppressors LKB1 and KEAP1, which have both been shown to drive immune evasion. LKB1 mutations are associated with an immune-excluded TME (44) and loss of LKB1 in KRAS-mutant mouse models drives immune evasion by promoting infiltration of neutrophils via IL6 (45) and suppressing tumor-intrinsic STING signaling (46), type I IFN signaling, and antigen presentation (47). Similarly, mutations in KEAP1 are associated with a T-cell excluded TME (48). KEAP1 loss leads to the accumulation of EMSY, which directly represses transcription of type I IFN response genes (49).

## Prospects for Combining KRAS Inhibition with Immunotherapy

The approval of immune checkpoint blockade (ICB) antibodies targeting the immunosuppressive PD-L1/PD-1 axis has revolutionized the treatment landscape for patients with KRAS-mutant LUAD (50, 51). These immunotherapies act to reinvigorate antitumor immune responses and can achieve durable responses in a subset of patients. KRAS G12C mutations are predominantly caused by smoking and are associated with a high-tumor mutational burden, which is predictive of response to ICB (52). However only a minority of patients with lung cancer respond to ICB and other KRAS-mutant cancers including PDAC and (MMR-proficient) colorectal cancer have shown little benefit (53).

**Figure 2.**
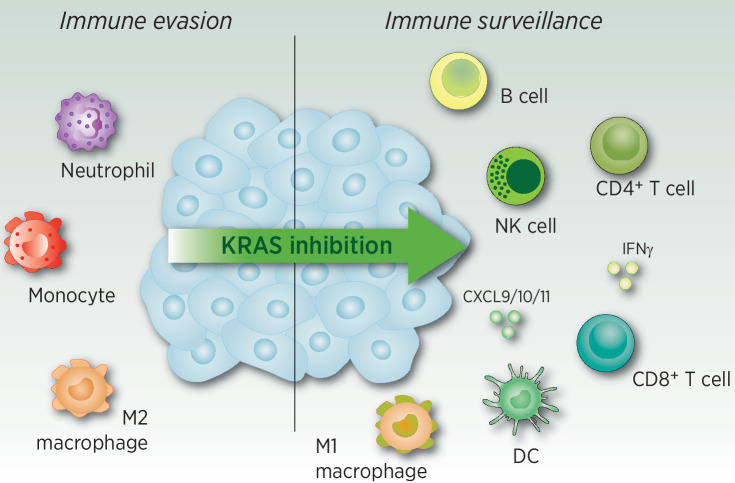
Mutant-specific KRAS inhibition remodels the immune TME. KRAS mutant-specific inhibition reverses KRAS-mediated immune suppressive mechanisms and results in a reduction of immunosuppressive cells and an increased recruitment of pro-inflammatory cells.

Given the established role of oncogenic KRAS in suppressing antitumor immunity there exists a strong rationale for combining KRAS inhibitors with immunotherapy. Indeed, numerous preclinical studies have shown that KRAS G12C inhibitors can at least partially reverse the KRAS-mediated immunosuppressive mechanisms described above and cause a profound remodeling of the TME in KRAS G12C-mutant lung and colorectal cancer (Fig. 2; refs. 12, 31, 54, 55). KRAS inhibition resulted in increased CD8^+^ T-cell influx and activation, polarization of the myeloid compartment, increased antigen presentation, and upregulation of IFN signaling transcriptional programs. The mechanisms underlying some of these changes have been partly elucidated and reveal a combination of direct tumor cell-intrinsic effects, such as decreased secretion of monocyte and neutrophil chemoattractants, and indirect effects, including stimulation of CXCL9 secretion by dendritic cells, which promotes infiltration of activated T cells (31). Intriguingly, KRAS G12C inhibition has also been shown to expand tertiary lymphoid structures (TLS; ref. 56), which are associated with immunotherapy response in multiple solid cancers (57), and boost the production of protective tumor-binding antibodies. However, the mechanism underlying this is still unknown. The novel KRAS G12D inhibitor, MRTX1133, has similarly been shown to enhance antitumor immunity in mouse models of PDAC (58). The ability of KRAS inhibition to stimulate antitumor immunity is also supported by conditional genetic mouse models of KRAS-mutant colorectal cancer (28) or PDAC (59) in which suppression of KRAS expression increased influx of T cells. Although the changes in immune cell populations and cytokine milieu in response to KRAS inhibition are well described, it is still unclear whether the pro-inflammatory effects are entirely due to the direct reversal of KRAS-mediated immunosuppression or also involves the induction of immunogenic cell death, which is important for the priming of antitumor immunity.

Strikingly, T cells and/or B cells are required for durable responses to KRAS inhibitors in mouse models of lung cancer, colorectal cancer, and PDAC (12, 54, 58), suggesting that the priming of an adaptive immune response may efficiently eliminate drug resistant cells. This is supported by preliminary clinical evidence that clinical resistance to AMG 510 in refractory tumors is associated with immune evasion (60). However, although KRAS inhibition as a monotherapy can generate complete responses in some immunogenic mouse models, durable responses have not been achieved in clinical settings. This inferior response is, in part, due to the extensive tumor heterogeneity that exists in late-stage human tumors and suggests further immunomodulatory interventions will be needed. Indeed, numerous studies have demonstrated the therapeutic benefits of combining KRAS inhibitors with ICB in KRAS-mutant mouse cancer models. Combination treatment with KRAS G12C inhibitor plus anti-PD-1 blockade cured the majority of mice in the CT-26 syngeneic colorectal cancer subcutaneous model (12, 55) and in a T-cell inflamed, TLS containing KRAS-mutant orthotopic LUAD model (31, 54), and also caused sustained tumor regression in an orthotopic PDAC model (bioRxiv. 2023.02.15.528757). Conversely, forced expression of oncogenic KRAS in the ICB-responsive KRAS wild-type MC38 model abrogated the activity of anti-PD-1 blockade (28). More preclinical models should be assessed to gain better insight into the efficacy of this combination across different cancer subtypes and baseline immune profiles, with a specific focus on orthotopic models which most accurately reflect the human disease and the tissue specific differences in the TME. Indeed, numerous studies have shown that subcutaneous and orthotopic tumors have different immune profiles and response to immunotherapy (54).

Studies with KRAS inhibitors mirror earlier work, which demonstrated the ability of BRAF and MEK inhibitors to enhance the therapeutic activity of ICB in LUAD, colorectal cancer, and melanoma mouse models (61–63). The combination of vemurafenib, cobimetinib, and atezolizumab has recently been approved by the FDA for the treatment of BRAF-mutant melanoma, which was shown in a phase III clinical trial to improve progression-free survival (64). However, the clinical utility of this combination is currently limited as the benefits are moderate and come at the cost of high toxicities. In fact, two other phase III trials of combination inhibition of BRAF, MEK, and PD-1 failed to show benefit in melanoma.

With regard to MEK inhibitors, along with other generalized blockers of MAPK signaling including ERK and pan RAS inhibitors, it should be noted that the RAS/MAPK pathway plays a critical and often multifaceted role in the regulation of most immune cells, for example being activated very rapidly upon both T-cell receptor and B-cell receptor stimulation. Any generalized inhibition of this pathway runs the risk of impairing immune cell function and hence compromising possible combination benefits between RAS pathway blockade and immunotherapy. For this reason, mutant selective KRAS inhibitors, which target KRAS only in the tumor cells and not the immune system, are expected to be more promising candidates for combination with immunotherapy than pan RAS, MEK, or ERK inhibitors. Alternatively, intermittent dosing of RAS pathway inhibitors may enable sufficient pathway inhibition in tumor cells, while maintaining immune cell activity as has been shown with the MEK inhibitor selumetinib (65).

Several clinical trials are currently testing the combination of KRAS inhibitors with ICB (Table 1). Whether this combination will prove more efficacious than either therapy alone remains unclear. However, more urgently, it is clear from Amgen's Codebreak 100/101 clinical trial that combining sotorasib and anti-PD-(L)1 antibodies result in greatly increased incidence of grade 3 to 4 liver toxicities (66). Increased liver toxicities have also been described in patients that received sotorasib after a previous treatment with anti-PD-1 therapies, probably reflecting the long half-life of anti-PD-1 antibodies (67, 68). This mirrors previous experiences in clinical trials combining targeted therapies with ICB including EGFR inhibitors (69)and ALK inhibitors in NSCLC (70).

**Table 1.** Clinical trials evaluating the combination of KRAS–G12C inhibitors with immune checkpoint inhibitors.

Drug	Combination	Clinical trial	Company	Phase	Disease setting	Comments
Sotorasib	Anti–PD-1/PD-L1	CodeBreak 100 (NCT03600883)	Amgen	Phase I/II	Advanced solid tumors	
Sotorasib	Pembrolizumab (PD-1) Atezolizumab (PD-L1)	CodeBreak 101 (NCT04185883)	Amgen	Phase I/II	Advanced solid tumors	
Adagrasib	Pembrolizumab (PD-1)	KRYSTAL-1 (NCT03785249)	Mirati Therapeutics	Phase 1/2	Advanced solid tumors	
Adagrasib	Pembrolizumab (PD-1)	KRYSTAL-7 (NCT04613596)	Mirati Therapeutics	Phase II/III	Advanced NSCLC, any PD-L1 TPS, candidate for first-line treatment	Includes phase III comparison with pembrolizumab plus chemotherapy
Adagrasib	Nivolumab (PD-1)	Neo-Kan (NCT05472623)	Mirati Therapeutics	Phase II	Resectable NSCLC	Neoadjuvant treatment for 6 weeks prior to surgery
Adagrasib	Pembrolizumab (PD-1)	NCT05609578	Mirati Therapeutics	Phase II	Advanced NSCLC with PD-L1 TPS ≥1%	Two cycles of adagrasib followed by combination
GDC-6036	Atezolizumab (PD-L1)	NCT04449874	Genentech	Phase I	Advanced solid tumors	
JDQ443	Tislelizumab (PD-1)-/+ TNO155 (SHP2i)	KontRASt-01 (NCT04699188)	Novartis	Phase I/II	Advanced solid tumors	
MK-1084	Pembrolizumab (PD-1)	NCT05067283	Merck	Phase I	Advanced NSCLC	
LY3537982	Pembrolizumab (PD-1)	NCT04956640	Eli Lilly	Phase I	Advanced solid tumors	
IBI351	Sintilimab (PD-1) ± chemotherapy	NCT05504278	Innovent Biologics	Phase I	Advanced nonsquamous NSCLC	

Note: All trials recruited patients harboring KRAS–G12C mutations.

The mechanism underlying these toxicities is still unclear and will require further investigation. One hypothesis is that the immune-modulatory effects caused by targeted therapies may enhance immune-mediate toxicities driven by immunotherapy. In this case, similar toxicities might be observed with other KRAS inhibitors. Another hypothesis is that the maximal dosing of sotorasib is causing off-target covalent protein–drug conjugates leading to liver damage, which is exacerbated by systemic immune activation due to ICB. Indeed, Genentech's KRAS G12C inhibitor GDC-6036, which is administered at lower doses, has shown less liver toxicities in phase I clinical testing (71). If this is the reason of the difference between both compounds, more potent inhibitors which require even lower doses could reduce the toxicities. In addition, patients receiving a lead-in dosing strategy or lower doses of sotorasib with ICB have shown less toxicities (66). Given this, Mirati has started a recent clinical trial (NCT05609578) based on a lead-in strategy which tests two different doses. Indeed, similar approaches have been used over the past decade in search of the optimal sequencing regiment for combining MAPK inhibitors with ICB in melanoma. Similar clinical trials assessing different treatment sequences of KRAS inhibitors and ICB, including intermittent scheduling or sequencing of combinations, may be required to maximize the therapeutic effect of combinations while minimizing toxicities. Furthermore, it is still unclear whether the observed increase in toxicities is specific to sotorasib or due to the covalent chemistry shared by other KRAS inhibitors. If this is the case, it is possible that such toxicities will not be observed with the recently developed KRAS G12D inhibitor MRTX1133, which is noncovalent. There is hope with the recent early reports from Mirati's KRYSTAL-7 clinical trial combining adagrasib with pembrolizumab which did not result in substantial high-grade liver toxicities, although patient numbers as yet remain small (72).

The ability of KRAS inhibition to enhance the efficacy of ICB is only seen in immunogenic tumor models, which are already partially responsive to immunotherapy and no such synergy is observed in intrinsically “cold” tumor models, which are immunotherapy refractory to start with (31). This observation has important clinical implications as it suggests only KRAS-mutant patients with an inflamed TME would benefit from the combination of KRAS inhibitors and ICB and calls into question whether such a combination would be effective in ICB nonresponsive cancer types such as colorectal cancer and PDAC. Moreover, different immunologic characteristics can also be observed across different KRAS mutations. For instance, in NSCLC, KRAS G12D mutations are associated with low or never smoking status, lower PD-L1 expression, and worse outcomes to PD-(L)1 blockade (73), which could suggest potential variations in response among different mutant-specific inhibitors. Importantly, the majority of clinical trials testing the combination of KRAS inhibition and ICB include mostly patients that have previously progressed after immunotherapy treatment and therefore the potential benefits of this combination may not be fully realized. KRYSTAL-7 is the only clinical trial currently evaluating the combination in ICB-naive patients and has demonstrated an impressive ORR (49%) with a subset of patients experiencing durable responses.

Furthermore, it is currently unclear whether common immunosuppressive co-occurring mutations, including LKB1 and KEAP1, may negatively impact the immunomodulatory effects of KRAS inhibition and its ability to enhance the response to ICB. Numerous clinical studies have demonstrated an association between co-mutations in LKB1 and KEAP1 with poor response to ICB in KRAS-mutant LUAD (74, 75). These patients may require additional targeted therapies to restore sensitivity to ICB. Preclinical studies have sensitized LKB1-mutant LUAD to ICB by inhibiting autophagy, which led to increased proteasome activity and antigen presentation (47) or Axl receptor tyrosine kinase inhibition, which enhanced type I interferon secretion in dendritic cells resulting in the expansion of ICB-responsive TCF1^+^ stem-like CD8^+^ T cells (76). Therefore, specific therapeutic approaches will need to be designed for patients with different comutations and baseline immune profiles.

## Future Directions

Clinical efforts are currently focused on assessing the combination of KRAS inhibition with anti-PD-L1/PD-1 antibodies. However, it is still unclear whether the issues of toxicity will be overcome and the extent of the clinical benefits. Therefore, novel strategies are also being explored to combine KRAS targeting with immunotherapies. Oncogenic mutations in KRAS generate clonal neoantigens that are presented by MHC-I, which can be recognized by cytotoxic T cells. The clinical benefits of this recognition were first realized in a case of metastatic colorectal cancer, which was successfully treated by adoptive T-cell therapy using *ex vivo* expanded tumor-derived lymphocytes that specifically recognized KRAS G12D (77). This observation paved the way for the development of therapeutic KRAS-specific T-cell receptors that bind mutant forms of KRAS with high specificity and affinity (78, 79), which are now being tested in clinical trials (80). Beyond cell-based therapies, numerous vaccines have also been developed to target KRAS-mutant tumor antigens. Moderna have developed a lipid nanoparticle-formulated mRNA vaccine (mRNA-5671/V941), which targets four of the most common oncogenic KRAS mutations (G12C, G12D, G12V, and G13D) and is currently being assessed in early-phase clinical trials in combination with pembrolizumab. This is a promising approach given the recent results of a phase I clinical trial in PDAC, which demonstrated responses in patients treated with an mRNA vaccine targeting personalized neoantigens (81). Alternatively, these therapies could also be used in combination with KRAS inhibitors to eliminate drug resistant or persister cancer cells.

Recent work has also demonstrated that covalent KRAS G12C inhibitors can be used to generate novel MHC-restricted drug–peptide neoantigens, which can be targeted by antibodies (82, 83). T-cell engagers (BiTE) have been generated from these antibodies, which can direct T-cell–mediated killing of KRAS G12C tumor cells. Such an approach may benefit from the combination of oncogenic KRAS signaling inhibition and immune recognition of tumor cells. Furthermore, KRAS G12C targeting BiTEs can also recognize tumor cells that are resistant to KRAS inhibition provided that drug engagement is maintained and therefore could be used to overcome targeted therapy resistance. Although great progress has been achieved in targeting KRAS-mutant tumor antigens, this approach is limited to a subset of patients as the mutant KRAS peptide is only presented on specific HLA alleles.

Preclinical studies exploring the response of tumors to KRAS G12C inhibitors using spatial and multi-omic technologies have highlighted the role of particular populations of immune cells in the TME in impeding immune attack on the tumor, pointing the way to novel approaches to combining RAS inhibition with immunotherapies. In one study, imaging mass cytometry revealed a dramatic remodeling of the TME in a “cold” model of lung cancer upon KRAS inhibition involving expansion of a macrophage subset found exclusively within the tumor domain, which interacted with fibroblasts and expressed markers of antigen-presentation including CD86 and MHC-II (84). Furthermore, this spatial analysis identified a distinct immune hub within tumors comprising dendritic cells, Tregs, and activated CD8 T cells. Another study employing deep profiling of preclinical models of PDAC by single-cell RNA-sequencing demonstrated a shift in macrophage phenotype upon KRAS inhibition characterized by depletion of an *Arg1^+^* cluster and enrichment of an *Mrc1^+^* cluster (bioRxiv. 2023.02.15.528757). Targeting specific immune populations, such as macrophages or Tregs, could therefore potentiate the immunomodulatory effects of KRAS inhibition, however it is currently unclear whether similar changes in the TME occur in human patients. Deep immune profiling of patients in response to KRAS inhibition, which can be assessed in the neoadjuvant setting, would increase our understanding of the mechanisms underlying therapy response and identify novel rational combination therapies. Interestingly, both Mirati and Amgen have started neoadjuvant trials in surgically resectable patients, providing a valuable opportunity to obtain tumor material for conducting these types of studies.

An alternative approach could be to target KRAS-driven immune evasion instead of KRAS. For example, oncogenic KRAS promotes resistance to ICB by inducing the secretion of CXCR2 ligands, which leads to the accumulation of myeloid-suppressor cells in the TME (28). Targeting CXCR2 has been shown to synergize with ICB in multiple preclinical models of KRAS-mutant colorectal cancer and PDAC (28, 30, 85), providing a potential combination therapy for the treatment of KRAS-mutant patients with cancer in the clinic. Similarly, the KRAS effector protein TBK1 has been identified by CRISPR screening as a mediator of immunotherapy resistance (86). A greater understanding of how oncogenic KRAS drives immune evasion and immunotherapy resistance will hopefully lead to additional rational ICB combination strategies that can be tested in the clinic and may avoid the toxicities currently associated with combining KRAS inhibitors with ICB.

Results from the recent phase III clinical trial assessing sotorasib in KRAS-mutant NSCLC demonstrate that adaptive resistance is common and occurs rapidly. Targeted combination therapies may therefore be required to enhance the efficacy of KRAS inhibitors to achieve more durable pathway inhibition. Such combinations may also prove more effective in potentiating antitumor immune responses. Several clinical trials are underway assessing the efficacy of KRAS inhibitors in combination with other targeted therapies including CDK4/6 and SHP-2 inhibitors. SHP-2 helps to mediate upstream activation of KRAS, especially the G12C mutant form that cycles more rapidly than most other mutants; however, it is also expressed in numerous immune cell types and inhibition of SHP-2 has been shown in preclinical tumor models to promote antitumor immunity and synergize with ICB (87). CDK4/6 drive cell-cycle progression downstream of the MAPK pathway and inhibition of CDK4/6 synergized with KRAS inhibition in xenograft models. Furthermore, CDK4/6 inhibitors have been shown to stimulate antitumor immunity by suppressing the proliferation of Tregs (88). As with the combination of KRAS inhibition and ICB, substantial effort will be required to understand how best to combine these additional KRAS targeting therapies with immunotherapy to minimize toxicities and maximize clinical benefits.

Although the initial excitement from the approval of sotorasib and adagrasib has died down due to the realization of their limitations in the clinic, there is a large body of evidence to support the hope that combining KRAS inhibitors with immunotherapies may be able to provide significantly improved outcomes in the treatment of KRAS mutant cancers. Early negative experiences with sotorasib combinations with PD-(L)1 blockade indicate that there will be many more hurdles to overcome and much more to learn about the underlying biology, but it is undeniable that there is huge potential in combining the orthogonal approaches of targeting KRAS signaling together with reactivating the immune system against the tumor. The next few years should show whether this promise can be realized and deliver major clinical benefit for patients.
